# Role of accelerated segment switch in exons to alter targeting (ASSET) in the molecular evolution of snake venom proteins

**DOI:** 10.1186/1471-2148-9-146

**Published:** 2009-06-30

**Authors:** Robin Doley, Stephen P Mackessy, R Manjunatha Kini

**Affiliations:** 1 Protein Science Laboratory, Department of Biological Sciences, National University of Singapore, 117543, Singapore; 2 School of Biological Sciences, University of Northern Colorado, Greeley, CO, 80639-0017, USA

## Abstract

**Background:**

Snake venom toxins evolve more rapidly than other proteins through accelerated changes in the protein coding regions. Previously we have shown that accelerated segment switch in exons to alter targeting (ASSET) might play an important role in its functional evolution of viperid three-finger toxins. In this phenomenon, short sequences in exons are radically changed to unrelated sequences and hence affect the folding and functional properties of the toxins.

**Results:**

Here we analyzed other snake venom protein families to elucidate the role of ASSET in their functional evolution. ASSET appears to be involved in the functional evolution of three-finger toxins to a greater extent than in several other venom protein families. ASSET leads to replacement of some of the critical amino acid residues that affect the biological function in three-finger toxins as well as change the conformation of the loop that is involved in binding to specific target sites.

**Conclusion:**

ASSET could lead to novel functions in snake venom proteins. Among snake venom serine proteases, ASSET contributes to changes in three surface segments. One of these segments near the substrate binding region is known to affect substrate specificity, and its exchange may have significant implications for differences in isoform catalytic activity on specific target protein substrates. ASSET therefore plays an important role in functional diversification of snake venom proteins, in addition to accelerated point mutations in the protein coding regions. Accelerated point mutations lead to fine-tuning of target specificity, whereas ASSET leads to large-scale replacement of multiple functionally important residues, resulting in change or gain of functions.

## Background

Snake venoms contain a mixture of proteins and polypeptides which exhibit various biochemical and pharmacological functions. These proteins and polypeptides are classified into non-enzymatic and enzymatic proteins which belong to a small number of superfamilies, such as three-finger toxins (3FTx), Kunitz-type serine protease inhibitors, phospholipase A_2_(PLA_2_) enzymes, serine proteases and metalloproteases [1-12]. Members of these superfamilies have similar protein scaffolds but, at times, differ markedly in their biological effects. For example, members of 3FTx family exhibit a wide variety of specific pharmacologic effects by targeting various receptors and ion channels with high affinity and specificity. Short chain and long chain α-neurotoxins antagonize muscle nicotinic acetylcholine receptors [5,12], κ-bungarotoxins recognize neuronal nicotinic receptors [13], muscarinic toxins are selective agonists/antagonists of distinct sub-types of muscarinic acetylcholine receptors [14], fasciculins inhibit acetylcholinesterase [15], calciseptine and related toxins block the L-type Ca^2+ ^channels [16,16,17], cardiotoxins/cytotoxins exert their toxicity by forming pores in cell membranes [18], and dendroaspins are antagonists of various cell-adhesion processes [19].

Similarly, other venom proteins, such as the Kunitz-type serine protease inhibitors, have a conserved fold and are structurally similar to bovine pancreatic trypsin inhibitor (BPTI) [20]. They have been reported to inhibit proteolytic activity of trypsin or chymotrypsin specifically [3,21,21-23]. In addition, some inhibitor-like proteins specifically block potassium and calcium channels [24-27]. Snake venom PLA_2 _isoenzymes, also characterized by a highly conserved fold, are known to induce various pharmacological activities such as neurotoxic, myotoxic, cardiotoxic, anticoagulant, and antiplatelet effects through specific interaction with their target proteins (for a review see [28]). Thus many subfamilies and isoforms of snake venom serine proteases and metalloproteases act on various components of the coagulation cascade and induce procoagulant or anticoagulant effects, as well as affect platelet aggregation, fibrinolytic and kallikrein-kinin systems [29-35]. The isoforms of the different superfamilies are known to evolve through a process of gene duplication followed by accelerated point mutations in the protein coding regions.

In venom proteins, comparisons of cDNA and gene sequences have shown that nonsynonymous nucleotide substitutions (leading to change in amino acid residues) are commonly greater than synonymous nucleotide substitutions (not producing change in amino acid residues) in the protein coding region compared to the non-coding (UTRs) and intron regions [36-38]. Thus, protein coding regions of genes encoding 3FTxs [39-41], Kunitz-type serine protease inhibitors [42], PLA_2 _enzymes [7,43,44] and serine proteases [8] appear to be undergoing accelerated point mutations, resulting in numerous isoforms. Individual point mutations affect one residue at a time, leading to small change in the surface characteristics of a protein. Therefore, point mutations may contribute to fine tuning of toxin specificities by (a) improving the specificity towards a particular receptor or ion channel; (b) altering the specificity towards a closely related receptor or ion channel; and (c) modifying the species specificity. However, accelerated point mutations may not be sufficient to explain drastic changes in the molecular surface needed for the observed targeting of toxins with conserved scaffolds to diverse receptors or ion channels.

In a recent study, we identified five transcripts encoding 3FTxs from the cDNA library of venom gland tissues of *Sistrurus catenatus edwardsii *[45]. These transcripts showed very low sequence similarity with elapid 3FTxs except for the conserved signal peptide and the number and position of cysteines. A systematic comparison of their sequences revealed that some of the segments in the mature proteins were 80–100% identical, whereas other segments were only 12.5–50% similar [46]. Some segments in the protein coding region seem to be exchanged with distinctly different segments, keeping the structural fold intact during their evolution. Interestingly, the segments in the introns of genes encoding these same proteins show high similarity (>85%) when present; and profound differences in segments appear to be restricted to exons only. Such switching of segments in the exon alters the surface topology and charge of the mature protein, which might alter the molecular targets of 3FTxs and contribute to the evolution of novel function. Therefore, we proposed that the phenomenon of **a**ccelerated **s**egment **s**witch in **e**xons to alter **t**argeting (ASSET) might play an important role in the evolution of 3FTxs in viperid snake venoms [46].

Here we have analyzed isoforms of 3FTxs from elapid snake venoms, as well as toxins from other protein superfamilies, to evaluate whether ASSET plays a role in their evolution. Elapid 3FTxs have been found to undergo changes due to ASSET as observed for viperid 3FTxs. Due to such exchange of segments, functionally important residues are changed, which might significantly affect their function. In some of these toxins, such change has lead to the evolution of demonstrated novel functions [19,47], and thus the 3FTx toxin family seem to be functionally evolving through ASSET. In the Kunitz-type serine protease inhibitor family, ASSET does not seem to play an important role in evolution, even though there are multiple isoforms. The enzymatic families, such as PLA_2 _and metalloproteases, appear to be evolving more through accelerated point mutations rather than ASSET. In these families, some of the segments seem to be exchanged during their evolution, but functional implication of such changes is not clearly understood. However, in the serine protease family, three segments near the substrate binding region have been found to be undergoing accelerated exchange of segments, and at least one of them may have significant implications for their substrate specificity.

## Results and Discussion

### Three-finger toxin (3FTx) family

3FTxs form a well-characterized superfamily of non-enzymatic proteins. They have a canonical three-finger fold of extending β- sheeted loops that is stabilized by four conserved disulphide bridges in the core region. Until recently, this family of proteins was thought to be present only in elapid venom [48]. However, 3FTxs have now been reported in colubrid venoms and in viperid venom gland transcriptomes as well [45,49-52]. Different isoforms of 3FTxs bind to various receptors/acceptors and exhibit diverse pharmacological functions despite their similar folding (for a review see [53]). Functionally important residues that are involved in interacting with the target receptors/ion channels generally reside in the tip of the loops [54]. Therefore the amino acid sequences, length and conformation of the loops play important role in their functional specificity (for reviews see [53,55].

**Figure 1:**
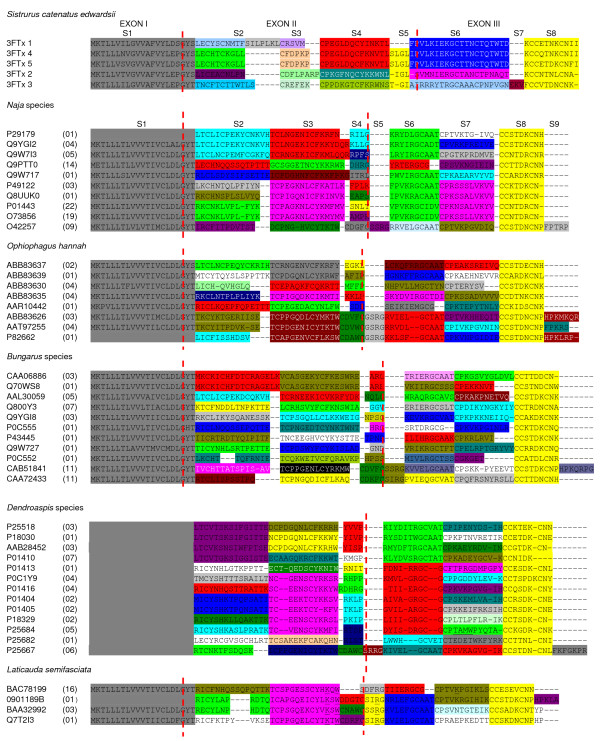
**Alignment of three-finger toxins from snake venoms**. Protein sequences were obtained from the NCBI database and are presented with their accession numbers. The signal peptide is similar in all the proteins and is shown in grey color; for those protein sequences whose signal peptide is not available, it is kept blank. The number of sequences of closely related homologues is shown in brackets. Based on structural identity, the mature protein is divided into several segments (S1–S9). To emphasize sequence similarities, segments with >50% identity are given similar colors, whereas <50% are given dissimilar colors. The gaps are inserted to optimize the alignments and shown as '-', and some of the functionally important amino acid residues are underlined (see text for details). The intron-exon boundary is marked with vertical red dashed lines.

Amino acid sequences of 3FTxs isolated from venoms of the same genus of elapids were grouped together and analyzed for ASSET and point mutations (Figure 1). The segments with high identity (60–100%) are shown in similar colors, whereas those with low identity (13%–50%) are shown in dissimilar colors. For example, among *Naja *3FTxs, the S2 segments in P29179, Q9YGI2 and Q9W713 show 60–100% identity (shown in turquoise), and the same segment shows 91.7% identity between O73856 and P01443 (shown in green). However, they share low identity with corresponding segments in other species (shown in various colors). Thus, *Naja *3FTxs have seven different types of S2 segments. Overall, there are nine (S1–S9) distinct segments (Figure 1) with varied identities, and within the same genus some segments are represented by more than one type. S1 (signal peptide) and S8 segments are the most conserved among all elapid 3FTxs, whereas S5 and S9 are found only in long chain 3FTxs. The replacement of segments has been found to occur only in exons II and III (exon-intron boundaries are marked by red dashed lines), while exon I codes for the highly conserved signal peptide in all 3FTxs. Thus, as in the case of *Sistrurus *3FTxs, elapid toxins also show discrete replacement of segments in protein coding regions. Such accelerated exchange of segments (ASSET) results in drastic changes in function (discussed below).

The three-finger folds of 3FTxs are held together by four conserved disulfide bridges. However, some 3FTxs have a fifth disulfide bridge in either the second loop (long chain neurotoxins and κ-neurotoxins [56]) or the first loop (non-conventional toxins [57]). The insertion of the fifth disulfide bridge in the second loop of long chain 3FTx is due to a change in the intron-exon boundary. This alteration in the intron-exon boundary is due to an insertion of a single nucleotide "A" in intron 2 which causes a shift in the splicing site [40], leading to the insertion of a short segment (S5) containing a cysteine residue. In the S4 segment, there is also a frame shift due to the deletion of a nucleotide, leading to a completely different sequence which also contains a new cysteine residue. Both cysteine residues form the fifth disulphide bridge and a cyclic structure in the second loop that is important to their binding to α7 receptors with high affinity [58]. The insertion of this short segment in long chain 3FTxs leads to a new function – binding to α7 receptors. In contrast, the fifth disulphide bridge in the first loop of short chain 3FTxs is due to exchange of segments (ASSET). This additional fifth disulphide bridge does not change the overall fold but it causes subtle changes in the first loop which are known to have functional implications [56]. Further, the number of amino acid residues in this segment differs among the toxins and hence would lead to change in the length of the loop.

**Figure 2:**
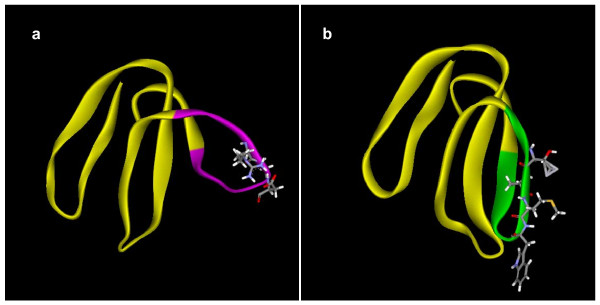
**Ribbon model of (a) dendroaspin (PDB ID**: 1DRS) **and (b) FS2 (**PDB ID: 1TFS). In dendroaspin, the segment CFTPRGDMPGPY is shown in magenta, whereas in FS2, the segment CPTAMWPYQTA is shown in green. Side chains of RGD and TAMW, the key residues in their functional motifs are shown. This segment exchange has profound effects on activity: dendroaspin is a potent antiplatelet protein interacting with α_IIb_β_3_, whereas FS2 is a potent blocker of L-type Ca^2+ ^channels.

As mentioned above, the loops play a crucial role in the recognition of target receptors/ion channels. For example, the third loop in dendroaspin (or mambin; P01413) contains the "RGD" tripeptide sequence (underlined in Figure 1) which is known to bind to platelet glycoprotein (GP IIb-IIIa; α_IIb_β_III_) and cause inhibition of platelet aggregation [19]. The S7 segment containing this sequence is replaced in other *Dendroaspis *toxins (Figure 1). This exchange of segment seems to be responsible for the loss of antiplatelet function in them. In one of the toxins (P25684), this segment is replaced with a segment containing the TAMW residues (underlined in Figure 1). In calciseptine, FS2 and other related toxins, this sequence is known to be involved in binding to L-type Ca^2+ ^channels [47]. This segment in dendroaspin and FS2 shows a different conformation (Figure 2) which might also influence their function. As shown in Figure 1, the S7 segment is the most variable among the *Dendroaspis *toxins and may result in functional diversification due to accelerated exchange.

Short chain and long chain α-neurotoxins are known to antagonize muscle nicotinic acetylcholine receptors, resulting in flaccid paralysis [5,12]. The structure-function relationships of α-neurotoxins have been thoroughly studied using both chemical modification and genetic engineering approaches [54,59-61]. Unlike dendroaspin and calciseptine (Figure 1), the functional site in the neurotoxins is discontinuous and is distributed on all three loops [54]. In erabutoxin a (BAC78199), the important functional residues involved in binding to *Torpedo *electroplax or to muscle nAChR (α_2_βγδ) are Lys^27^, Trp^29 ^(S3), Asp^31^, Phe^32^, Arg^33 ^(S5) and Lys^47 ^(S7) (underlined in Figure 1) [56]. Although Lys^27^, Trp^29 ^and Arg^33 ^are conserved in all *Laticauda *toxins, the other critical residues (Asp^31^, Phe^32 ^and Lys^47^) are replaced via exchange of segments. We hypothesize that these segment exchanges may have a direct impact on their ability to bind to *Torpedo *or muscle (α_2βγδ_) receptors.

It is also important to note that there are minor changes in amino acid residues within the identical segments (highlighted in white in Additional file 1) and these changes are due to an accelerated rate of point mutations. Both ASSET and accelerated point mutations have contributed to the functional diversity of elapid 3FTXs; ASSET leads to major changes in the surface properties, resulting in targeting of new receptors, while accelerated point mutations lead to fine-tuning of binding to the same receptors through minor alterations of the surface charge and topology.

### Kunitz-type serine protease inhibitor family

Snake venom Kunitz-type serine protease inhibitors have been reported from both elapid and viperid venoms. Structurally, they are similar to Kunitz/BPTI inhibitors with a conserved fold stabilized by three disulphide bridges [6]. As with other toxin families, the isoforms are encoded by a multigene family and have evolved through gene duplication and positive selection [8]. The isoforms from the same genus are grouped together as above and analyzed for ASSET and accelerated point mutations (Additional file 2).

Though the snake venom Kunitz-type serine protease inhibitor family contains multiple isoforms, functionally they are not as diverse as other venom protein superfamilies, and they can be divided into either non-neurotoxic or neurotoxic homologs. Non-neurotoxic homologs inhibit either trypsin or chymotrypsin, while neurotoxic homologs act as calcium and potassium channel blockers which do not have protease inhibitory activity [26,27,42]. Structurally, both groups have a conserved fold similar to BPTI, but the inhibitor binding loops and the β turn regions have undergone adaptive evolution, resulting in new biological activities [6]. Analysis of the amino acid sequences of the isoforms shows that there is no radical change in the amino acid residues in the mature proteins, as observed in 3FTxs. However, they have undergone adaptive evolution through accelerated point mutation (Additional file 2). In calcicludine and dendrotoxin-I, the N-terminal part and overall conformation play a significant role in calcium and potassium channel-blocking activity (Additional file 2). This has been demonstrated by synthesizing chimeras containing the N-terminal (1–30) of calcicludine and C-terminal (31–60) of dendrotoxin-I, and vice versa [24]. However, there are not enough Kunitz-type serine protease inhibitors and dendrotoxin sequences from *Dendroaspis *species in the database in order to determine if they have evolved through ASSET. Similarly, the B chain of β-bungarotoxin (from *Bungarus*) is also a Kunitz-type serine protease inhibitor but does not have protease inhibitory activity; however, it contributes to neurotoxicity [62]. The interaction of the B chain with the potassium channel was predicted to be localized opposite of the anti-protease loop, between residues 27–30 [63]. In addition to this, the mature protein shows accelerated point mutations which resulted in the introduction of a cysteine residue at the C-terminal end (underlined in Additional file 2). This extra cysteine residue forms the disulphide bridge with chain A [63]. Further, the C-terminal region of chain B shows a conformational change due to its interaction with the chain A and accounts for the lack of protease inhibitor activity [63]. Unlike 3FTxs, where ASSET has played an important role in the evolution of new functions, deviation of some members of Kunitz-type serine protease inhibitors from protease inhibitory activity is mainly due to accelerated point mutations. This might explain the low functional diversity in this group of toxins, even though they have multiple isoforms.

### Phospholipase A_2 _(PLA_2_) family

PLA_2 _enzymes are one of the best-studied hydrolytic enzymes and are found abundantly in nature. Snake venoms are a good source of these enzymes and often contain multiple isoenzymes. In addition to a role in the digestion of prey, they induce a wide variety of pharmacological effects in prey/victims (for a review see [64]). It has been well documented that accelerated point mutations have occurred in the protein coding regions, and this adaptive mode of evolution might also be responsible for acquisition of new functions [65]. We analyzed the elapid and viperids PLA_2 _isoenzymes to determine if ASSET has played any role in the functional evolution of these toxins.

**Figure 3:**
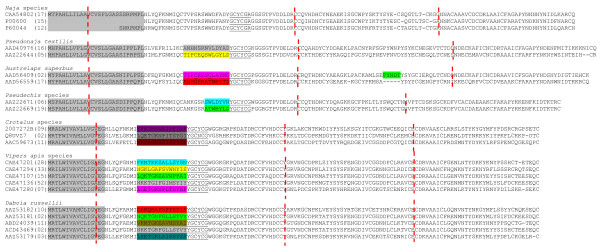
**Alignment of phospholipase A_2 _isoforms from snake venoms**. Protein sequences were obtained from the NCBI database and are presented with their accession numbers. The signal peptide is highlighted in grey color. The number of sequences of closely related homologues is shown in brackets. The calcium binding loop is underlined and gaps are shown with "-". The region undergoing exchange of segment is given different color coding.

**Figure 4:**
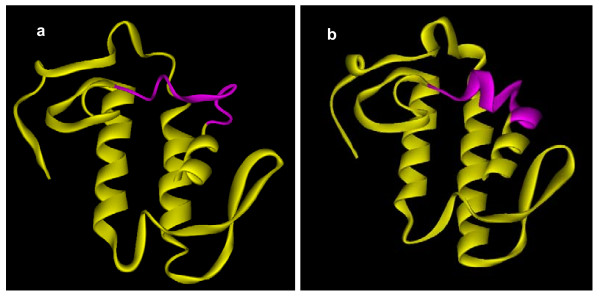
**Ribbon model of (a) daboiatoxin (PDB ID**: 2H4C) **and (b) Vipera PLA_2 _(PDB ID**: 1Q6V) **showing conformational change due to segment exchange**. These changes may impart differences in biological function (not yet evaluated).

Comparison of the amino acid sequences of PLA_2 _isoenzymes in all genera revealed that the N-terminal region seems to be undergoing exchange of segment except in *Naja *species (Figure 3; segments are shown in different colors). This segment is 13–14 amino acids long (forms helix B in several PLA_2 _enzymes) and lies between the first helix and the calcium-binding loop. Differences in exchange within the same species seem to arise due to ASSET, as there is more than one amino acid replacement. As this segment lies near the calcium-binding region, it might affect catalysis due to changes in the conformation in this region. For example, in viperid PLA_2 _enzymes, the sequence LEETGKLAIPSYSS (in AAZ53179) is replaced with an unrelated sequence VKMTGKEAVHSYAI (in ABD24039), which clearly imparts significant conformational changes (Figure 4). However, the impact of such replacements on catalysis is not clear. In elapids within the same genera, this segment is represented by two different types, but in viperids there are several types. Interestingly, in AAD56409 and the γ subunit of taipoxin, as a result of this replacement, two cysteine residues are inserted. These additional cysteines form the extra disulphide bridge, in addition to the seven conserved disulphide bridges of PLA_2 _enzymes [66]. Further, the residues in this short segment have been proposed to play crucial role in some of the pharmacological effects of PLA_2 _enzymes [67]. Ammodytoxin A from the venom of *Vipera ammodytes ammodytes *interacts with human FXa through surface residues distributed in helices and the calcium binding loop. One of the helices (helix B), which is undergoing accelerated segment exchange, is involved in this interaction [67]. Replacement of critical amino acid residues through segment exchange can influence the biological activity of PLA_2 _enzymes. Other than this segment, the mature protein in all the groups appears to be evolving via accelerated point mutations. Though elapid and viperid PLA_2 _enzymes have evolved from different lineages, it is interesting to note that the exchange of the segment in both groups occurs at the same position. Such an exchange may impart conformational and/or functional changes.

In addition to this exchange of a segment near the calcium binding region, the presence or absence of another segment has been observed in exon III of *Austrelaps superbus *PLA_2 _enzymes (Figure 3, shown in green color). This segment represents the pancreatic loop, an ancestral feature found in the pancreatic PLA_2 _enzymes. Pancreatic PLA_2 _enzymes show low hydrolytic activity due to the presence of this loop, and the deletion of this loop in porcine PLA_2 _results in 16 times higher catalytic activity [68]. Thus, the deletion of pancreatic loop in venom PLA_2 _enzymes plays an important role in the evolution of catalytically more active enzymes.

Accelerated point mutations in the mature protein of PLA_2 _enzymes are known to play important roles in functional evolution [37,43,69,70]. These substitutions appear to occur mostly in the surface residues and thus alter the specificity of targeting to various tissues or cells, resulting in distinct pharmacological effects [71]. Though we observed ASSET near the calcium-binding region, its role in functional evolution of PLA_2_s is not yet clear.

### Serine protease family

Snake venom serine proteases (SVSPs) are one of the well characterized families of enzymes that affect the hemostatic system. They act on various components of the coagulation cascade, fibrinolytic and kallikrein-kinin systems as well as on platelets to cause significant perturbance of the haemostatic system [31,72-75]. This family of enzymes are believed to have evolved from glandular kallikrein and trypsin-like enzymes, as they have similar gene structure and share common three-dimensional structure [76]. Similar to other multigene families, they have evolved through accelerated evolution in the protein coding region [8]. In the present study we aligned the SVSPs from *Trimeresurus *species, *Crotalus *species, *Sistrurus catenatus edwardsii *and *Bothrops jararaca *obtained from the database to analyze for ASSET.

**Figure 5:**
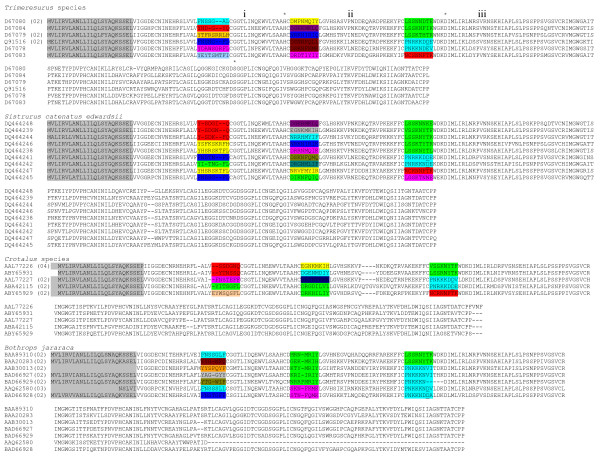
**Alignment of serine protease isoforms from snake venoms**. Protein sequences were obtained from the NCBI database and are presented with their accession numbers. The signal peptide is highlighted in grey color. The number of sequences of closely related homologues is shown in brackets. The segments undergoing exchange are marked as i, ii and iii and are given different color coding. The active site residues are marked with asterisks.

**Figure 6:**
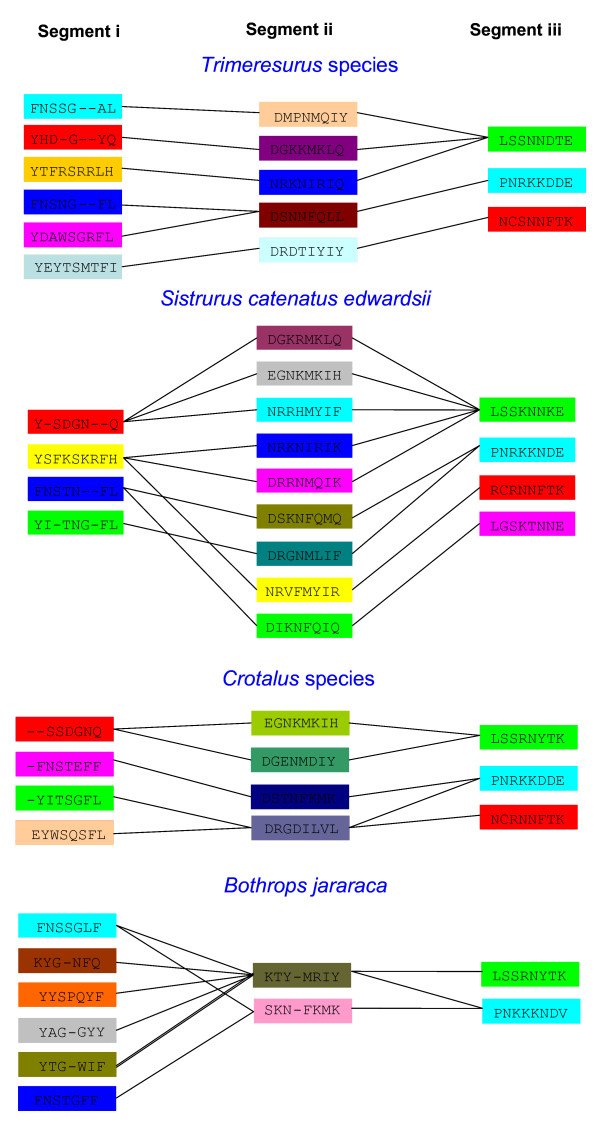
**Diagrammatic representation of the segment i, ii and iii with amino acid sequence**. The pattern of existence of segments in the isoforms demonstrates that they do not follow any similar pattern but are random.

Comparison of the isoenzymes among the same group reveals that the signal peptide is highly conserved in all the groups, and the mature protein shows accelerated evolution. Particularly, three segments in the mature proteins seem to be undergoing accelerated exchange. These segments are identified in all the different genera and named as i, ii and iii (Figure 5; shown in different colors). Among them, segment iii is the most conserved segment and is represented by only four different types (shown in green, light blue, red and magenta, in Figure 5) across the genera whereas segments i and ii exist in several different forms. These segments were further analyzed to see if a similar pattern exists across different genera (Figure 6). Although some similar patterns emerge, the data strongly supports accelerated exchange of these segments. Further, the segment exchanges appear to be random and we could not link them through simple molecular phylogeny.

**Figure 7:**
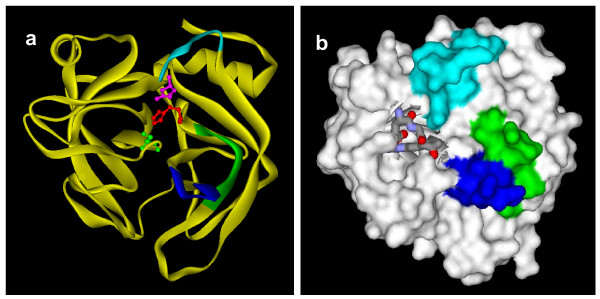
**Ribbon (a) and surface (b) models of plasminogen activator (TSV-PA) (PDB ID**: 1BQY). The segments that undergo exchange are shown in green, dark blue and turquoise color. The side chains of the active site residues are shown in the ribbon model. The substrate binding area is shown in turquoise color.

It is interesting to note that all three segment exchanges occur on the surface of the protein (Figure 7) which is important for substrate binding and interactions with ligands. SVSPs are all characterized by the presence of a highly reactive serine residue (Ser) in the active site. In addition, His57 and Asp102 present in the active cleft are important for catalysis (marked with an asterisk in Figure 6); this active cleft is located at the junction of two six-stranded β-barrels [77]. The entrance to this active site cleft is influenced by several surface loops of the protein. Asp97, present in segment iii, is the most important residue in substrate recognition, and a D97N mutant shows markedly decreased substrate binding capacity [78]. This residue (D97 in Q91516 (PSV-PA) is underlined in Figure 5. However, in other isoforms from *Trimeresurus*, this segment PNRKKDDE (shown in turquoise color) is replaced with either NCSNNFTK (shown in red color) or LSSNNDTE (shown in green color) with the loss of critical Asp residue. Thus ASSET may have a direct impact on the substrate binding of these isoforms. On the other hand, changes of segment i and ii might not influence the catalytic activity, as they are away from the active cleft and are not involved in substrate binding (Figure 7). However, they might still contribute to targeting to various tissues or proteins as they are fully exposed on the surface of these enzymes. This family of proteins seems to be evolving via both ASSET and accelerated point mutations.

### Metalloprotease family

**Figure 8:**
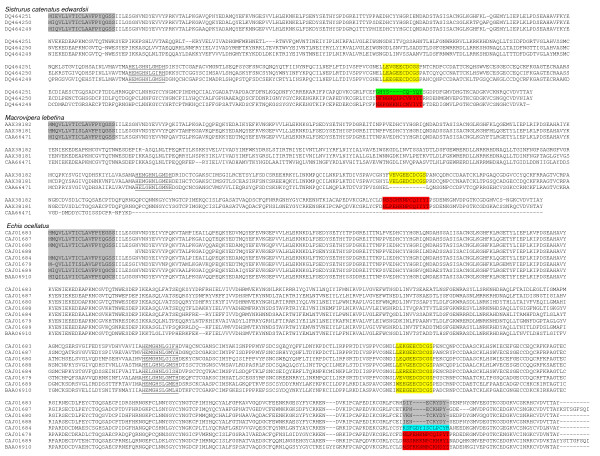
**Alignment of metalloprotease isoforms from snake venoms**. Protein sequences were obtained from the NCBI database and are presented with their accession numbers. The signal peptide is highlighted in grey color and the zinc binding site is underlined. The number of sequences of closely related homologues is shown in brackets. Segments undergoing exchange are shown in different colors.

**Figure 9:**
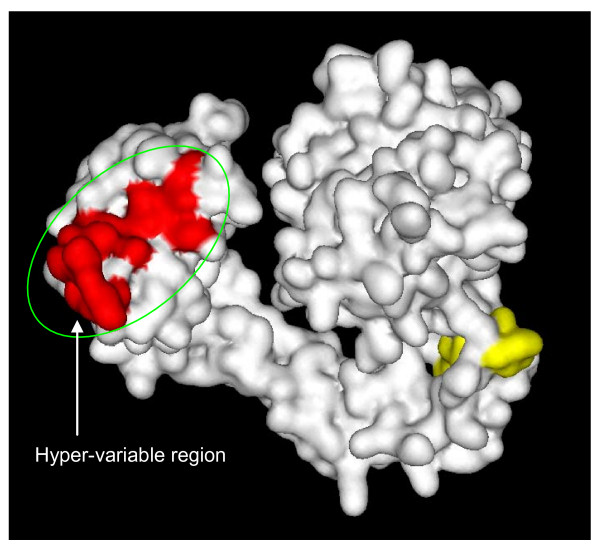
**Surface model of catrocollastatin/vascular apoptosis-inducing protein (VAP) 2B (PDB ID**: 2DW1) **showing exchange of segments**. Surface area shown in red is the hyper-variable segment found to be undergoing exchange in other metalloproteases whereas the yellow colored region is the shoulder domain.

Snake venom metalloproteases (SVMPs) are the main toxic components present in the venom of many Viperidae [2,11]. They are synthesized as zymogens containing multidomain precursors and undergo proteolytic processing. Structurally, they are categorized into four types, PI, PII, PIII and PIV, based on the presence of different domains and their quaternary structure [9,79]; the metalloprotease domain is present in all the subtypes. Catalytically it is the most important domain and contains the zinc binding site with a consensus sequence of HEXXHXXGXXH [80]. This catalytic domain is known to play an important role in inducing hemorrhage during envenomation and is highly conserved [81]. SVMPs induce hemorrhage at the site of the bite by cleaving proteins in the basement membrane and subsequently weakening capillaries [82]. At present, not many isoforms of metalloproteases have been reported from the same species. Sequences from *Sistrurus catenatus edwardsii*, *Macrovipera lebetina *and *Echis ocellatus *were obtained from the database and analyzed for point mutations and ASSET (Figure 8). Most of the residues in the mature protein seem to be conserved, except for a few residues which have been replaced through accelerated point mutations. However, in the isoforms of *Sistrurus catenatus edwardsii *venom, the cysteine-rich domain seems to be undergoing exchange of segments (shown in red/green colors). Similarly, in the spacer region between the metalloprotease and the disintegrin-like domains of *Macrovipera lebetina *isoforms, 11 amino acid residues have been deleted in one of the isoforms (Figure 8). These two regions were analyzed in the crystal structure of catrocollastatin/vascular apoptosis-inducing protein (VAP) 2B from *Crotalus atrox *venom, which possesses metalloproteinase/disintegrin/cysteine-rich (MDC) domains (Figure 9). The segment represented by VGEECDCGTPE is a part of the shoulder domain containing one of the calcium binding regions, and this calcium binding region is absent in one of the isoforms of *Macrovipera lebetina *metalloprotease. The other segment, highlighted with red color, is the most variable and divergent among the ADAM/adamalysin/reprolysin protein family and is known as hyper-variable region. This hyper-variable region has been predicted to be a potential exosite for target recognition [83]. This segment in one of the isoforms of *Sistrurus catenatus edwardsii *has been replaced with another segment (highlighted in green color) and hence might recognize a different substrate or lose its substrate binding property.

## Molecular mechanism of ASSET

Previously, we discussed the possible molecular mechanisms of ASSET, including splicing variation, recombination, accumulation of point mutations and independent recruitment events [46]. Nevertheless, we believe that none of these explanations are satisfactory. Splicing variations, such as alternative splicing and changes in the splicing site, can lead to insertion/deletion of alternate segments in the mature protein. However, all but one segment change occur within the exons and not at the intron-exon boundaries. In the long chain 3FTxs only, the insertion of a segment occurs at the intron-exon boundary due to a shift in the splicing site (discussed above). Genetic recombination might also give rise to replacement of segments in the mature protein. However, the segment exchanges observed in the venom proteins are too small, and canonical recombination processes cannot explain exchange of short segments. The possibility of accumulation of point mutations producing the observed change in segments cannot be ruled out unequivocally, as venom proteins have been well-documented to evolve through accelerated point mutations [7,8,37,39-43,84]. In such circumstances, this would have to occur over many generations to attain the observed change in segments, and intermediates would have to be selected via positive selection. Further, the same point mutations would have to occur independently in several unrelated lineages to produce the same segment composition. Thus, odds are against the accumulation of point mutations as an explanation. The 3FTxs showing these exchanges could have occurred through independent recruitment events, but significant similarities in protein sequence and gene structure (particularly high similarity among intron sequences) show that they have evolved from a common ancestor. The observed changes in protein segments could also be due to insertion/deletion of 1–2 nucleotides, resulting in a frame shift and hence altered protein sequence. In such a case, a similar number of nucleotide(s) must be removed/added in a downstream region, respectively, to get back into the original open reading frame. Therefore, we carefully analyzed each of these segment exchanges at the nucleotide level. Only one segment, RKCHNSPLSLVYQ (S2 in Q8UUK0), is changed to RKCNKLVPL-FYK in P01443 (Figure 1A) due to deletion and insertion of nucleotides. In this case, there is an insertion of A at the 78^th ^position (from the start codon) and a deletion of four nucleotides downstream (Additional file 3). Therefore, none of the above possibilities explain the observed exchange of segments in the exons. It is important to note that in spite of the segment exchanges, the cysteine residues, which maintain the three dimensional fold, are conserved in 3FTxs. Although the molecular mechanism of the exchange of segments is not yet understood, these events clearly play a significant role in the functional evolution of some snake venom proteins.

## ASSET occurs at the molecular surface

Surface residues of a protein molecule are important for their physicochemical properties as well as for their interactions with biomolecules, including other proteins. Accordingly, the alteration of the conformation and surface properties indeed affects the pharmacological properties of protein toxins. In an earlier paper, we showed that in PLA_2 _enzymes the surface residues have undergone natural substitution 2.6–3.5 times faster than the buried residues and proposed that accelerated point mutations preferentially target the surface residues in PLA_2 _enzymes, leading to the evolution of new isoforms with distinct functions [71]. As shown here, ASSET also targets surface residues in 3FTXs, PLA_2 _enzymes, serine proteases and metalloproteases (Figure 2, 4, 7 and 9). Accelerated point mutations result in finer modifications to the surface topology and/or electrostatic potential, whereas ASSET drastically alters the surface, essentially instantaneously producing large-scale changes in the ligand interaction site(s). The molecular mechanisms of both accelerated point mutation and ASSET are not clearly understood, but both phenomena play a crucial role in the evolution of snake venom proteins.

## Conclusion

Elapid 3FTxs, similar to viperid 3FTxs (Doley et al., 2008), evolve by both ASSET and accelerated point mutations. ASSET affects the entire mature protein of 3FTxs except for segment S8, which is highly conserved. In serine proteases, three of the surface segments are changed rapidly by ASSET, but the rest of the mature protein evolves only by accelerated point mutations. In PLA_2 _enzymes and metalloproteases, only one and three surface segments (respectively) are changed via ASSET. In all these superfamilies of toxins, ASSET most likely affects their functional properties. However, serine protease inhibitors have evolved by only accelerated point mutations. We propose that ASSET occurs first, resulting in drastic changes in functionally important surface regions, followed by accelerated point mutations in those regions which fine-tune the target specificity. Although the molecular mechanisms of ASSET and accelerated point mutations are unknown, both contribute to the evolution of snake venom toxins and both help to explain the observed functional diversity of toxins and the evolution of new functions in snake venom protein superfamilies.

## Methods

### Sequence analysis and identification of segments

The protein and cDNA sequences were obtained from the NCBI database. Sequence alignments were done using the DNAMAN program and by manual examination. The intron-exon boundary (marked by a red dashed line in the figures) was identified by comparing the gene and their respective cDNA sequences. In those toxins whose gene structure is not available, the intron-exon boundary was identified by comparing with other toxins whose boundary is known. These segments are identified by comparing with the corresponding sequences, and the point of deletion in amino acid sequence was identified as the boundary of most of the segments. We have analyzed the sequences in different species, but there is no absolute trend in defining the segment, other than high sequence identity. Color coding was used to distinguish segments with distinct % identity; segments with >50% identity are shown in the same color, whereas segments with <50% identity are shown in different colors. Ribbon and surface models were generated from PDB files using DS ViewerPro software.

## Abbreviations used

ASSET: Accelerated Segment Switch in Exon to alter Targeting.

## Authors' contributions

RD carried out the sequence analysis and drafted the manuscript. SPM participated in sequences analyses and writing of the manuscript. RMK designed the study, participated in sequence analyses and writing of the manuscript. All authors read and approved the final manuscript.

## Supplementary Material

Additional file 1 **Three-finger toxins from *Naja *species showing point mutations**. The segments undergoing exchanges are given different color coding. The residues showing point mutations are shown in white and red type whereas those that are conserved are in black. The gaps are represented by "-". This demonstrates accelerated point mutations occur in various segments and the segment exchange is not due to these point mutations. Similarly three-finger toxins from other snake venoms as well as toxins from other families show point mutations through out various segments (data not shown). Click here for file

Additional file 2 **Alignment of Kunitz-type serine protease inhibitor isoforms from snake venoms**. Protein sequences were obtained from the NCBI database and are presented with their accession numbers. Numbers of similar sequences are shown in brackets. They appear to evolve by accelerated point mutations in the mature protein (changes in residues are shown in red) but not by segment exchange. Click here for file

Additional file 3 **Segment S2 (Figure **1) **of three-finger toxins Q8UUK0 and P01443**. Figure shows deletion and addition of nucleotides (red color) in the segment. Click here for file
