# Supplementary material for: Role of accelerated segment switch in exons to alter targeting (ASSET) in the molecular evolution of snake venom proteins
Source: BMC Evol Biol. 2009 Jun 30;9:146. doi: 10.1186/1471-2148-9-146 (PMC2711939; doi:10.1186/1471-2148-9-146)
Supplement: Additional file 1 — Three-finger toxins from Naja species showing point mutations. The segments undergoing exchanges are given different color coding. The residues showing point mutations are shown in white and red type whereas those that are conserved are in black. The gaps are represented by "-". This demonstrates accelerated point mutations occur in various segments and the segment exchange is not due to these point mutations. Similarly three-finger toxins from other snake venoms as well as toxins from other families show point mutations through out various segments (data not shown). [file 1471-2148-9-146-S1.pdf]

Additional file1: Three-finger toxins from *Naja* species showing point mutations. The residues showing point mutations are shown in white and red type whereas those that are conserved are in black.

```
AAA49386                         LTCLICPEKYCNKVHTCLNGENICFKRFN-----RILGKRYDLGCAATCP-TVKTG-IVQCCSTDKCNH-------
0706221A                         LTCLICPEKYCNKVHTCLNGEKICFKRYSER---KLLGKRYIRGCADTCP-VRKPREIVQCCSTDKCNH-------
AAB25735                         LTCLNCPEVYCRRFQKCRNGEKICFKKFDQR---NLLGKRYEIGCAATCP-EAKPREIVQCCSTDKCNR-------
764177A                          LTCLNCPEVYCRRFQICRDGEKICFKKFDQR---NLLGKRYRRGCAATCP-EAKPREIVQCCSTDKCNR-------
O93422     MKTLLLTLVVVT-IVCLALGYTLTCLICPEKYCNKVHTCLNGEKICFKKYDQR---KLLGKRYIRGCADTCP-VRKPREIVECCSTDKCNH-------
CAA04578   MKTLLLSLVVVT-IVCLDLGYTLTCLNCPEMFCGKFQICRNGEKICFKKLHQR---RPFSLRYIRGCAATCP-ETKPRDMVECCSTDRCNR-------
P60814     MKTLPLTLVVVT-IVCLDLGYTLTCLNCPEMFCGKFQICRNGEKICFKKLHQR---RPFSLRYIRGCAATCP-GTKPRDMVECCSTDRCNR-------
Q802B3     MKTLLLTLVVVT-IVCLDLGYTLTCLNCPEMFCGKFQTCRNGEKICFKMLQQR---RPFSLRYIRGCAATCP-GTKPRDMVECCSTDRCNR-------
O42255     MKTLLLTLVVVT-IVCLDLGYTLTCLNCPEMFCGKFQTCRNGEKICFKKLQQR---RPFSLRYIRGCAATCP-GTKPRDMVECCSTDRCNR-------
Q9W7I3     MKTLLLTLVVVT-IVCLDLGYTLTCLNCPEMFCGKFQTCRNGEKICFKMLQQR---RPFSLRYIRGCAATCP-GTKPRDMVECCNTDRCNR-------
P60773                           LECHNQQSSQAPTTKTCSG-ETNCYKKWWS----DHRGTIIERGCG--CPKVKPGVKLN-CCRTDRCNN-------
Q9YGJ6                           LECHDQQSSETPTTTGCSGGETNCYKKSWR----DHRGYRIERGCG--CPSVKKGIEIN-CCTTDRCNN-------
P60774                           LECHNQQSSQAPTTKTCSG-ETNCYKKWWS----DHRGTIIERGCG--CPKVKPGVKLN-CCTTDRCNN-------
AAD09179                         MECHNQQSSQAPTTKTCSG-ETNCYKKWWS----DHRGTIIERGCG--CPKVKPGVNLN-CCTTDRCNN-------
AAD09180                         MECHNQQSSQTPTTTGCSGGETNCYKKWWS----DHRGTIIERGCG--CPKVKPGVNLN-CCTTDRCNN-------
AAA66026                         LECHDQQSSQAPTTTGCSGGETNCYKKSWR----DHRGYRIERGCG--CPSVKKGIEIN-CCTTDRCNN-------
ABY61977                         LECHDQQSSQTPTTTGCSGGETNCYKKRWR----DHRGYRTERGCG--CPSVKNGIEIN-CCTTDRCNN-------
P29182                           LECHNQQSSQPPTTKTCSG-ETNCYKKWWS----DHRGTIIERGCG--CPKVKPGVNLN-CCRTDRCNN-------
P80958     MKTLLLTLLVVT-IVCLDLGYTLECHNQQSSQTPTTKTCSG-ETNCYKKWWS----DHRGTIIERGCG--CPKVKPGVNLN-CCTTDRCNN-------
AAD08812   MKTLLLTLLVVT-IVCLDLGYTLECHNQQSSETPTTTGCSGGETNCYKKSWR----DHRGYRIERGCG--CPSVKKGIEIN-CCTTDRCNN-------
AAD08813   MKTLLLTLLVVT-IVCLDLGYTLECHNQQSSQAPTTTGCSGGETNCYKKSWR----DHRGYRIERGCG--CPSVKKGIEIN-CCTTDRCNN-------
AAC69916   MKTLLLTLLVVT-IVCLDLGYTLECHNQQSSQAPTTTGCSGGETNCYKKGWR----DHRGYRIERGCG--CPSVKKGIEIN-CCTTDRCNN-------
AAB01538   MKTLLLTLLVVT-IVCLDLGYTLECHNQQSSQTPTTTGCSGGETNCYKKRWR----DHRGYRTERGCG--CPSVKNGIEIN-CCTTDRCNN-------
AAF21774   METLLLTLLVVT-IVCLDLGYTLECHNQQSSQTPTTTGCSGGETNCYKKRWR----DHRGYRTERGCG--CPSVKNGIEIN-CCTTDRCNN-------
CAB45156   MKTLLLSLVVVT-IVCLDLGYTRLCLSDYSIFSETIEICPDGHNFCFKKFPKG---ITRLPWVIRGCAATCPKAEARVYVD-CCARDKCNR-------
CAC85538   MKTLLLTLVVVT-IVCLDLGYTRKCHN--SPLSLVYQTCPIGQNICFKINVK----EAPSIPVKRACAATCPKSSALVKVV-CCKTDKCN--------
CAA90967   MKTLLLTLVVVT-IVCLDLGYTLKCHN--TQLPFIYNTCPEGKNLCFKATLK----FPLKFPVKRGCAATCPRSSSLVKVV-CCKTDKCN--------
AAB36928   MKTLLLTLVVVT-IVCLDLGYTLKCHN--TQLPFIYKTCPEGKNLCFKATLKK---FPLKFPVKRGCADNCPKNSALLKYV-CCSTDKCN--------
AAB36929   MKTLLLTLVVVT-IVCLDLGYTLKCHN--TQLPFIYKTCPEGKNLCFKATLKK---FPLKFPVKRGCADNCPKNSALLKYV-CCSTEKCN--------
P25669                           LKCN---KLIPLAYKTCPAGKNLCYKMFMV----AAPKVPVKRGCIDACPKNSLLVKYV-CCNTDRCN--------
P60309                           LKCK---KLVPLFSKTCPPGKNLCYKMFMV----AAPKVPVKRGCINVCPKSSLLVKYV-CCNTDKCN--------
P60308                           LKCK---KLVPLFSKTCPPGKNLCYKMFMV----ATPKVPVKRECIDVCPKSSLLVKYV-CCNTDKCN--------
```

```
AAB33650                          LKCN---KLVPLFYKTCPAGKNLCYKMFMV----ATPKVPVKRGCIDVCPKSSLLVKYV-CCNTDRCN--------
CAA69977  MKTLLLTLVVVT-IVCLDLGYTLKCN---QLIPPFYKTCAAGKNLCYKMFMV----AAQRFPVKRGCIDVCPKSSLLVKYV-CCNTDRCNN-------
CAA90965  MKTLLLTLVVVT-IVCLDLGYTLKCN---QLIPPFYKACAAGKNLCYKMFMV----AAPKVPVKRGCIDVCPKSSLLVKYV-CCNTDRCS--------
CAB42058  MKTLLLTLVVVT-IVCLDLGYTLKCN---QLIPPFYKTCAAGKNLCYKMFMV----AAPKVPVKRGCIDVCPKSSLLVKYV-CCNTDRCN--------
CAA90966  MKTLLLTLVVAT-IVCLDLGYTLKCN---QLIPPFYKTCAAGKNLCYKMFMV----AAPKVPVKRGCIDVCPKSSLLVKYV-CCNTDRCN--------
CAB41507  MKTLLLTLVVVVTIVCLDLGYTLKCN---QHIPPFYKTCAAGKNLCYKIFMV----AAPKVPVKRGCIDVCPKSSDLVKYV-CCNTDRCN--------
AAB33649  MKTLLLTTVVVT-IVCLDLEYTLKCN---KLVPLFYKTCPAGKNLCYKMFMV----ATPKVPVKRGCIDVCPKSSLLVKYV-CCNTDRCN--------
AAC27686  MKTLLLTLVVVT-TVCLDLGYTLKCN---KLVPLFYKTCPAGKNLCYKMYMV----ATPKVPVKRGCIDVCPKSSLLVKYV-CCNTDRCN--------
P60303    MKTLLLTLVVVT-IVCLDLGYTLKCN---KLVPLFYKTCPAGKNLCYKMFMV----ATPKVPVKRGCIDVCPKSSLLVKYV-CCNTDRCN--------
AAC27686  MKTLLLTLVVVT-TVCLDLGYTLKCN---KLVPLFYKTCPAGKNLCYKMYMV----ATPKVPVKRGCIDVCPKSSLLVKYV-CCNTDRCN--------
CAA90963  MKTLLLTLVVVT-IVCLDLGYTLKCN---KLVPLFYKTCPAGKNLCYKMFMV----ATPKVPVKRGRIDVCPKSSLLVKYV-CCNTDRCN--------
CAB42055  MKTLLLTLEEET-IVCLDLGYTLKCN---KLVPLFYKTCPAGKNLCYKMFMV----ATPKVPVKRGCIDVCPKSSLLVKYV-CCNTDRCN--------
AAC27687  MKTLLLTLVLVT-IVCLDLGYTLKCN---KLVPLFYKTCPAGKNLCYKMYMV----ATPKVPVKRGCIDVYPKSSLLVKYV-CCNTDRCN--------
AAB18383  MKTLLLTLVVVT-IVCLDLGYTLKCN---KLVPLFYKTCPAGKNLCYKMFMV----ATPKVPVKRGCIDVCPKNSLLVKYV-CCNTDRCN--------
AAC27689  MKTLLLTLVVVT-IVCLDLGYTLKCN---KLVPLFYKTCPAGKNLCYKMFMV----AMPKVPVKRGCIDVCPKSSLLVKYV-CCNTDRCN--------
AAC27690  MKTLLLTLVVVT-IVCLDLGYTLKCN---KLVPLFYKTCPAGKNLCYKMYMV----AMPKVPVKRGCIDVCPKSSLLVKYV-CCNTDRCN--------
AAA66026                          LKCN---KLVPLFYKTCPAGKNLCYKMFMV----SNKMVPVKRGCIDVCPKSSLLVKYV-CCNTDRCN--------
P01446                            LKCN---KLIPLAYKTCPAGKNLCYKMFMV----SNKTVPVKRGCIDVCPKNSLLVKYV-CCNTDRCN--------
P60306                            LKCN---KLKPLAYKTCPAGKNLCYKMFMM----SNKTVPVKRGCIDVCPKNSLLVKYV-CCNTDRCN--------
P25668                            LKCN---KLIPLAYKTCPAGKNLCYKMFMV----SNKTVPVKRGCIDVCPKNSLVLKYV-CCNTDRCN--------
P01440                            LKCN---KLIPLAYKTCPAGKNLCYKMYMV----SNKTVPVKRGCIDVCPKNSLVLKYE-CCNTDRCN--------
AAC61318                          LKCN---KLVPLFYKTCPAGKNLCYKMFMV----SNKTVPVKRGCIDVCPKNSALVKYV-CCNTDRCN--------
P01391                            LKCN---KLIPLAYKTCPAGKNLCYKMFMV----SNKTVPVKRGCIDACPKNSLLVKYV-CCNTDRCN--------
AAC61318                          LKCN---KLVPLFYKTCPAGKNLCYKMFMV----SNKTVPVKRGCIDVCPKNSALVKYV-CCNTDRCN--------
AAB86637                          RKCN---KLVPLFYKTCPAGKNLCYKMFMV----SNLTVPVKRGCIDVCPKNSALVKYV-CCNTDRCN--------
P01443    MKTLLLTLVVVT-IVCLDLGYTRKCN---KLVPLFYKTCPAGKNLCYKMFMV----SNLTVPVKRGCIDVCPKNSALVKYV-CCNTDRCN--------
P01442    MKTLLLTLVVVT-IVCLDLGYTLKCN---KLVPLFYKTCPAGKNLCYKMFMV----SNLTVPVKRGCIDVCPKNSALVKYV-CCNTDRCN--------
P60310    MKTLLLTLLVVT-IVCLDLGYTLKCN---KLVPLFYKTCPAGKNLCYKMFMV----SNLTVPVKRGCIDVCPKNSALVKYV-CCNTDRCN--------
P01442    MKTLLLTLVVVT-IVCLDLGYTLKCN---KLVPLFYKTCPAGKNLCYKMFMV----SNLTVPVKRGCIDVCPKNSALVKYV-CCNTDRCN--------
Q9W6W9    MKTLLLTLVVVT-IVCLDLGYTRKCN---KLVPLFYKTCPAGKNLCYKMFMV----SNLTVPVKRGCIDVCPKSSLLVKYV-CCNTDRCN--------
CAA90962                          LKCN---KLVPIAYKTCPEGKNLCYKMFMM----SDLTIPVKRGCIDVCPKNSLLVKYV-CCNTDRCN--------
CAA90964                          LKCN---KLIPIASKTCPAGKNLCYKMFMM----SDLTIPVKRGCIDVCPKSNLLVKYV-CCNTDRCN--------
P01441                            LKCN---KLVPIAYKTCPEGKNLCYKMFMM----SDLTIPVKRGCIDVCPKNSLLVKYV-CCNTDRCN--------
P60311                            DKCN---KLVPLFYKTCPAGKNLCYKMFMV----SDLTVPVKRGCIDVCPKNSALVKYV-CCNTDRCN--------
Q91135    MKTLLLTLAAAT-IVCLDLGYTLKCN---KLIPIASKTCPAGMNLCYKMFMM----SDLTIPVKRGCIDVCPKNSLLVKYV-CCNTDRCN--------
Q91136    MKTLLLTLVVVT-IVCLDLGYTLKCN---KLIPIASKTCTAGKNLCYKMFMM----SDLTIPVKRGCIDVCPKNSLLVKYV-CCNTDRCN--------
```

```
P79810     MKTLLLTLVVVT-IVCLDLGYTLKCN---KLIPIASKTCPAGKNLCYKMFMM----SDLTIPVKRGCIDVCPKNSHLVKYV-CCNTDRCN--------
CAA90962   MKTLLLTLVVVT-IVCLDLGYTLKCN---KLIPIASKTCPAGKNLCYKMFMM----SDLTIPVKRGCIDVCPKNSLLVKYV-CCNTHRCN--------
P25673                          ITCY--KTPIPITSETCAPGQNLCYTKTWCDAWCGSRGKVIELGCAATCPTVESYQDIK-CCSTDDCNPHPKQKRP
P01382                          IRCF--ITP-DITSKDCPNG-HVCYTKTWCDAFCSIRGKRVDLGCAATCPTVKTGVDIQ-CCSTDNCNPFPTRKRP
P01427                          IRCF--ITP-DITSKDCPNG-HVCYTKTWCDAFCSIRGKRVDLGCAATCPTVKTGVDIQ-CCSTDDCDPFPTRKRP
P29180                          IRCF--ITP-DITSKDCPNG-HVCYTKTWCDGFCRIRGERVDLGCAATCPTVKTGVDIQ-CCSTDDCDPFPTRKRP
P29181                          IRCF--ITP-DITSKDCPNG-HVCYTKTWCDGFCSRRGERVDLGCAATCPTVKTGVDIQ-CCSTDDCDPFPTRKRP
P25672                          IRCF--ITP-DITSKDCPNG-HVCYTKTWCDGFCSSRGKRVDLGCAATCPTVRTGVDIQ-CCSTDDCDPFPTRKRP
P25671                          IRCF--ITP-DITSKDCPNG-HVCYTKTWCDGFCSIRGKRVDLGCAATCPTVRTGVDIQ-CCSTDDCDPFPTRKRP
CAA90966                        IRCF--ITP-DITSKDCPNG-HVCYTKTWCDGFCSSRGKRVDLGCAATCPTVRTGVDIQ-CCSTDDC-PFPTRKRP
AAL87469   MKTLLLTLVLVT-IMCLDLGYTIRCF--ITP-DVTSTDCPNG-HVCYTKTWCDGFCSSRGRRVELGCAATCPTVKPGVDIQ-CCSTDNCNPFPTR--P
```
