# Supplementary material for: Role of accelerated segment switch in exons to alter targeting (ASSET) in the molecular evolution of snake venom proteins
Source: BMC Evol Biol. 2009 Jun 30;9:146. doi: 10.1186/1471-2148-9-146 (PMC2711939; doi:10.1186/1471-2148-9-146)
Supplement: Additional file 2 — Alignment of Kunitz-type serine protease inhibitor isoforms from snake venoms. Protein sequences were obtained from the NCBI database and are presented with their accession numbers. Numbers of similar sequences are shown in brackets. They appear to evolve by accelerated point mutations in the mature protein (changes in residues are shown in red) but not by segment exchange. [file 1471-2148-9-146-S2.pdf]

Additional file 2: Alignment of Kunitz-type serine protease inhibitor isoforms from snake venoms. Numbers of similar sequences are shown in brackets. They appear to evolve by accelerated point mutations in the mature protein (changes in residues are shown in red) but not by segment exchange.

Daboia russellii

```
ABD24043        MSSGGLLLLLGLLTLWAELTPISGQDRPKFCHLPVDSGICRAHIPRFYYNPASNQCQGFIYGGCGGNANNFETRDQCRHTCGGK
ABD24042        MSSGGLLLLLGLLTLWAELTPISGHDRPKFCYLPADPGECMAYIRSFHYDSESKKCKEFIYGGCHGNANNFPTRDKCRQTCRGK
ABD24040        MSSGGLLLLLGLLTLWAELTPISGHDRPTFCNLAPESGRCRGHLRRIYYNLESNKCKVFFYGGCGGNANNFETRDECRQTCGGK
```

Bungarus species

```
AAL30069        MSSGGLLLLVGLLTLCAELTPVSSKDRPKFCNVPPEPGRCNANVRAFYYNPRLRKCIEFTYGGCGGNANNFKSGGECKRACGE--
AAL30068        MSSGGLLLLLGLLTLCAELTPVSSKDRPKFCNVPPEPGRCNANVRAFYYNPRLRKCIEFTYGGCGGNANNFKSRGECKRTCAE--
AAL30070        MSSGGLLLLLGLLTLWTELTPVSSKNRPPFCNLLPEPGRCNAIVRAFYYNSRLRKCLEFPYGGCGGNANNFKTIDECQRTCAG--
CAA72809  (5)  MSSGGLLLLLGLLTLCAELIPVSSRQRHRDCDKPPDKGNCGPVRRAFYYDTRLKTCKAFQYRGCNGNGNHFKTETLCRCECLVYP
CAJ18319  (5)  MSSGGLLLLLGLLTLCAELTPVSSRKRHPDCDKPPDTKICQTVVRAFYYKPSAKRCVQFRYGGCNGNGNHFKSDHLCRCECLEYS
BAC77654  (5)  MSSGSLLLLLGLLTLLAELTPVSSRKRHPDCDKPPNKKRCTGHIPAFYYNPQRKTCERFSYGGCKGNGNHFKTPQLCMCHCHE—
```

Naja species

```
CAE51866 (02)  MSSGGLLLLLGLLTLWAELTPVSGRPRFCELAPSAGSCFAFVPSYYYNQYSNTCHSFTYSGCGGNANRFRTIDECNRTCVG
P20229                                  RPGFCELPAAKGLCKAHKPAFYYNKDSHRCQKFIYGGCGGNANRFRTIDECNRTCVG
P19859   (02)                           RPRFCELAPSAGSCFGFVSSYYYNRYSNTCHSFTYSGCGKNANRFRTIDECNRTCVV
```

Pseudonaja textilis

```
ACC77787 (02)  MSSGGLLLLLGLLTLWEVLTPVSSKDRPELCELPPDTGPCRVRFPSFYYNPDEQKCLEFIYGGCEGNANNFITKEECESTCAA
AAK95519        MSSGGLLLLLGLLTLWEVLTPVSSKDRPDFCELPADTGPCRVRFPSFYYNPDEKKCLEFIYGGCEGNANNFITKEECESTCAA
ACC77788 (02)  MSSGGLLLLLGLLTLWEVLTPVSSKDRPKFCELLPDTGSCEDFTGAFHYSTRDRECIEFIYGGCGGNANNFKTLEECESTCAA
AAK95522        MSSGGLLLLLGLLTLWEVLTPVSSKDHPKFCELPADTGSCKGNVPRFYYNADHHQCLKFIYGGCGGNANNFKTIEECKSTCAA
AAK95348        MSSGGLLLLLGLLTLWEVLTPVSSKDRPKFCELPADIGPCDDFTGAFHYSPREHECIEFIYGGCKGNANNFNTQEECESTCAA
```

Demansia vestigiata

```
ABK63551 (02)  MSSGGLLLLLGLLTLWAELTPVSSKDRPEFCELPPDRGTCMGFLQAFYYNPSQNKCLPFMFGGCKANPNNFKTLEECKRTCAA
ABM86984        MSSGGLLLLLGLLTLWAELTPVSSKDRPEFCELPPDRGTCMGFLQAFYYNPSQKGCLPFMFGGCKANPNNFKTLEECKRTCAA
ABK63552        MSSGGLLLLLGLLTLWAELTPVSSKDRPEFCELPPDRGTCMGYSQAFYYNPSQNKCLPFMFGGCKANPNNFKTLEECKRTCAA
ABK63553        MSSGGLLLLLGLLTLWAELTPVSSKDLPEICKLPKEPGPCRSYLLYFYYNSVEHKCQTFHYGGCEGNENRFHTIEECKSTCAE
ABK63554        MSSGGLLLLLGLLTLWEVLTPVSSTDRPEFCELPEDSGPCKGLFHVFYYNSDQNQCLEFIYGGCYGNANNFKAIEECKRTCAA
```

Dendroaspis species

```
AAB29942 (Calcicludine)                          QPPWYCKEPVRIGSCKKQFSSFYFKWTAKKCLPFLFSGCGGNANRFQTIGECRKKCLGK
AAB26998 (Dendrotoxin K)SGHLLLLLGLLTLWAELTPVSGAAKYCKLPLRIGPCKRKIPSFYYKWKAKQCLPFDYSGCGGNANRFKTIEECRRTCVG
```
