# Supplementary material for: Role of accelerated segment switch in exons to alter targeting (ASSET) in the molecular evolution of snake venom proteins
Source: BMC Evol Biol. 2009 Jun 30;9:146. doi: 10.1186/1471-2148-9-146 (PMC2711939; doi:10.1186/1471-2148-9-146)
Supplement: Additional file 3 — Segment S2 (Figure 1) of three-finger toxins Q8UUK0 and P01443. Figure shows deletion and addition of nucleotides (red color) in the segment. [file 1471-2148-9-146-S3.pdf]

Additional file 3: Segment S2 (Figure 1) of three-finger toxins Q8UUK0 and P01443 showing deletion and addition of nucleotides (red color) in the segment.

```
AGGAAATGTCACAA-CTCACCACTTTCACTAGTCTATCAGACTTCTCCA
AGGAAATGTAACAAACTCGTTCCTTT----ATTCTATAAGACTTCTCCA
```
